# Supplementary material for: STAT1-IFITM3 promotes autophagy in epithelial cells to control Cryptosporidium parvum infection
Source: Life Sci Alliance. 2025 Jul 18;8(9):e202503200. doi: 10.26508/lsa.202503200 (PMC12274651; doi:10.26508/lsa.202503200)
Supplement: Supplementary file 1 [file LSA-2025-03200_Supplemental_Data_1.docx]

**Calculation of cell numbers and *C. parvum* growth**

The procedure used to measure and calculate the *C. parvum* infection burden is briefly described as follows. (1) HCT-8 cells were seeded and grown in a 96-well cell culture plate until they reach ~90% confluence. (2) Five-fold serial dilutions of *C. parvum* oocysts were made in 300 μl of culture medium for three replicates of 100 μl suspensions, each containing 1 × 10^1^, 1 × 10^2^, 1 × 10^3^, 1 × 10^4^, and 1 × 10^5^ oocysts per 100 μl. (3) The culture medium was removed from the cell culture plate and 100 μl of oocyst suspensions were added into individual wells. (4) The plate was placed into an incubator to allow the parasite oocysts to undergo excystation and invasion into host cells for 4 h at 37 °C. (5) The culture medium was replaced at 4 hours post-infection (hpi) with 200 μl/well of fresh medium to remove free parasites. The infected cells were incubated for an additional 20 h. (6) Cell lysates were prepared and qRT-PCR was performed. (7) The mean ∆C_T_ values were plotted from technical and biological replicates against the logarithm of the numbers of inoculated oocysts. (8) Linear regression analysis was carried out to obtain the standard curve, slope value, and R^2^ value to determine the detection efficacy and data coefficient. (9) The absolute oocyst counts were calculated based the on cycle threshold (C_T_) values and standard curves. (10) The relative parasite loads or inhibition of the parasite growth were calculated based on the C_T_ values of Cp-18S and Hs-18S transcripts. (11) The mean C_T[Cp18S]_ and C_T[Hs18S]_ values were calculated from the technical replicates for each biological replicate. (12) The level of Cp18S was normalized to that of Hs18S in each sample by computing the ∆C_T_ between CT_[Cp18S]_ and C_T[Hs18S]_. (13) The mean of ∆C_T_ values derived from all replicates of the negative control group in each plate or for each set of experimental condition were calculated. (14) The relative levels between individual samples and the mean of negative control were determined by computing the ∆∆C_T_ values. (15) The comparative threshold cycle (2^−ΔΔCt^) was used to evaluate the relative *C. parvum* burden of each simple.
